# Evaluation of Cyclosaplin Efficacy Using a Silk Based 3D Tumor Model

**DOI:** 10.3390/biom9040123

**Published:** 2019-03-28

**Authors:** Abheepsa Mishra, Sourav K. Mukhopadhyay, Satyahari Dey

**Affiliations:** 1 Plant Biotechnology Laboratory, Department of Biotechnology, Indian Institute of Technology Kharagpur, Kharagpur 721302, West Bengal, India; mukherjeesour@gmail.com (S.K.M.); sdey12.iitkgp@gmail.com (S.D.); 2 Department of Internal Medicine, The University of Texas Southwestern Medical Center, 5323 Harry Hines Blvd, Dallas, TX 75390, USA

**Keywords:** breast cancer, cyclosaplin, 3D tumor model, peptide, sandalwood, silk

## Abstract

Development of novel anti-cancer peptides requires a rapid screening process which can be accelerated by using appropriate in vitro tumor models. Breast carcinoma tissue is a three-dimensional (3D) microenvironment, which contains a hypoxic center surrounded by dense proliferative tissue. Biochemical clues provided by such a 3D cell mass cannot be recapitulated in conventional 2D culture systems. In this experiment, we evaluate the efficacy of the sandalwood peptide, cyclosaplin, on an established in vitro 3D silk breast cancer model using the invasive MDA-MB-231 cell line. The anti-proliferative effect of the peptide on the 3D silk tumor model is monitored by alamarBlue assay, with conventional 2D culture as control. The proliferation rate, glucose consumed, lactate dehydrogenase (LDH), and matrix metalloproteinase 9 (MMP-9) activity of human breast cancer cells are higher in 3D constructs compared to 2D. A higher concentration of drug is required to achieve 50% cell death in 3D culture than in 2D culture. The cyclosaplin treated MDA-MB-231 cells showed a significant decrease in MMP-9 activity in 3D constructs. Microscopic analysis revealed the formation of cell clusters evenly distributed in the scaffolds. The drug treated cells were less in number, smaller and showed unusual morphology. Overall, these findings indicate the role of cyclosaplin as a promising anti-cancer therapeutic.

## 1. Introduction

Three-dimensional (3D) in vitro models are currently inevitable in cancer biology as a bridge connecting two-dimensional (2D) cultures and the complexity associated with in vivo models. The war against cancer has gained momentum, as it is one of the major causes of death in the world and its causation is yet to be solved completely. In 2D cell cultures, the cancer cells grow in a monolayer fashion in a nutrient and oxygen enriched microenvironment with minimal contact to the surrounding cells. Apart from this, 2D cell culture models have other serious differences in cellular processes, metabolism, signaling, and response to anticancer drugs [1,2]. This leads to the low predictability of these 2D models in preclinical trials [3]. Thus, the 2D cell cultures fail to furnish an ideal environment for screening novel anticancer compounds. Initially, the traditional spotlight for development of chemotherapeutics was on tumor cells only. Lack of clinical efficacy and unacceptable toxicity are two of the main causes of failures during drug development [4]. The experimental studies revealed that synergy with the stroma in 3D systems might have an impact on sensitivity and resistance to drugs [5]. This highlights the importance of the microenvironment in designing and screening the efficacy of new peptide-based anticancer drugs. In contrast to 2D cell culture systems, the cancer cells in 3D cell cultures communicate with each other in a nutrient-oxygen poor microenvironment and recapitulate in vivo tumor conditions. Thus, 3D cell culture experiments serve as a convenient platform for probing the above cellular processes [6,7]. The extracellular matrix (ECM), considered as an essential component of the stroma, was shown to stimulate signal transduction pathways that might be absent when the drugs were screened in 2D flasks, showing this approach to be ineffective on tumors [8]. This could lead to failure in clinical trials. An in vitro tumor model is a vital tool for evaluating therapeutic efficacy of a drug before performing in vivo experiments. The advancement of translatable drug delivery systems clinically requires intensive evaluation of their tumor targeting potential in vitro, therapeutic efficiency, cytotoxic nature, and biocompatibility [9]. Thus, to imitate the in vivo characteristics of cancerous human tissue and testing the efficacy of peptide-based anticancer drugs, a 3D model needs to be used. The cancer cells in 3D cell culture systems adopt different morphology and cell growth, show increased glycolysis, and show a difference in anticancer drug sensitivity [10].

A 3D tumor model’s microenvironment can influence the topology and physiology of cancer cells in vivo, distinguishing them from cancer cells grown in 2D cell culture by several factors. One of the factors that can affect the tumor microenvironment is metabolism of the cancer cells. Tumor cell metabolism is one of the important attributes of cancer biology and recently, it has attained new heights. The cancer cell’s metabolism is altered compared to normal cells and this offers an edge for their survival and growth [11]. In cancer, the metabolism preferably shifts towards the glycolytic pathway (glucose to lactate) with a decreased dependency on the oxidative pathway (pyruvate to lactate to acetyl-CoA) [11,12]. This phenomenon is also known as the Warburg effect. This effect is enhanced in 3D cell culture models, providing an ideal microenvironment for cancer cell growth, progression, and pathogenesis. 

Several factors are involved in cancer pathogenesis, of which matrix metalloproteinases (MMPs) are extensively studied. MMP expression has been found to be upregulated in practically all types of advanced stage cancers in humans with invasive and metastatic potential and a poor prognosis [13,14,15]. Among the various MMPs, MMP-9 is the major enzyme for degradation of type IV collagen, a main component of the ECM. It also plays a vital role in tumor angiogenesis by enhancing vascular endothelial growth factor (VEGF) production [16]. Therefore, there is a growing interest in developing 3D in vitro models for testing the efficacy of anticancer drugs. Both natural and synthetic matrices have been developed for culture of cancer cells [17].

Silk, a natural polymer, is used as a multifaceted biomaterial in various forms, such as films, membranes, gels, sponges, powders, scaffolds [18], and nanoparticles [19,20]. The proliferation of human MDA-MB-231 cells on a silk fibroin 3D model has been recently reported [21]. The 3D silk fibroin model was used to study the efficacy of a few anticancer drugs, such as ZD6474, celexocib, and paclitaxel [22]. In our previous work we identified, purified, and structurally characterized a new cyclic octapeptide, cyclosaplin, from somatic seedlings of *Santalum album* L. [23], and to further assess its efficacy, a 3D-based silk tumor model was employed. Although there have been a few studies of silk-fibroin models to study the tumor microenvironment and screen anticancer drugs [2], there are no reports of this silk fibroin model being used to screen new cyclic peptides against breast cancer. In this study, the silk fibroin-based 3D in vitro tumor model was used for evaluating the efficacy of the novel cyclic peptide, cyclosaplin, prior to its in vivo application.

## 2. Materials and Methods

### 2.1. Materials

AlamarBlue (Molecular Probes, Invitrogen, Carlsbad, CA, USA), cellulose dialysis tubing of cut off 12,000 (Pierce, Puyallup, WA, USA), Dulbecco’s Modified Eagle’s Medium (DMEM) (Gibco, Invitrogen, Carlsbad, CA, USA), penicillin/streptomycin (Himedia, Mumbai, India), fetal bovine serum (Gibco), Gelatin (Sigma-Aldrich, St. Louis, MO, USA), Glucose Assay and Lactate assay Kit (Span Diagnostics, Surat, India), Live-Dead assay kit (Molecular Probes, Invitrogen, Carlsbad, CA, USA), 3-4,5-Dimethylthiazol-2-yl-2,5-Diphenyltetrazolium Bromide (MTT) (Himedia, Mumbai, India), Sodium dodecyl sulfate (SDS) (Pierce, Puyallup, WA, USA), tissue culture grade polystyrene flasks and cell culture plates (Tarsons, Kolkata, India), and trypsin-EDTA (Himedia, Mumbai, India) were used.

### 2.2. Preparation of Fibroin from *A. mylitta* Silkworm

*Antheraea mylitta* (*A. mylitta*) silk fibroin was fabricated as described in earlier reports [21,24]. The fifth instar mature larvae of *Antheraea mylitta* were dissected to collect the posterior glands. The glands were repeatedly rinsed in distilled water for removing the traces of sericin and squeezed to obtain fibroin protein. The fibroin protein was dissolved in 1% (*w*/*v*) SDS aqueous solution comprising 10 mM Tris (pH 8.0) and 5 mM EDTA at room temperature [21]. The solubilized protein was dialyzed with molecular weight cut off (MWCO) 12,000 against deionized water for regeneration of the 2% (*w*/*v*) aqueous protein solution. The fibroin solution was passed through a 0.22 μm filter before casting into molds for matrix fabrication.

### 2.3. Fabrication of 2D and 3D Silk Matrices

The silk fibroin (2% *w*/*v*) isolated from *A.mylitta* was used to coat the wells of 96 well tissue culture plates (TCP). The plates were kept overnight for drying, and the films were washed with 70% ethanol followed by phosphate buffered saline (PBS). The films were sterilized for 20 min by ultraviolet (UV) treatment. The silk fibroin film-coated tissue culture plates were used in cell culture experiments. For 3D matrices, the silk fibroin solution was poured into plates, frozen at −20 °C for 8 h and lyophilized, resulting in porous silk fibroin scaffolds [24]. Both the 2D and 3D silk matrices were stabilized by β-sheet formation induced by brief alcohol (absolute ethanol) treatment. The silk fibroin 3D scaffolds were washed in PBS and UV sterilized prior to cell culture studies.

### 2.4. Culture, Maintenance and Seeding of MDA-MB-231 Cells

MDA-MB-231 cells were sub-cultured in Dulbecco’s modified Eagle’s medium (DMEM) containing 10% fetal bovine serum and 1% penicillin G-streptomycin at 37 °C in a 5% CO_2_ humidified environment. At confluence, the cells were treated with Trypsin/EDTA to form a suspension, pelleted and finally re-suspended in fresh medium for cell seeding. Before cell culture, the silk fibroin matrices were sterilized by consecutive treatment with 70% ethanol and UV light for 30 min. Silk constructs were then washed thrice with sterile PBS (pH 7.4) and conditioned with complete medium for 4 h. Just before cell seeding, the matrices were partially dehydrated for 2 h to ensure proper cell permeability. Approximately 1 × 10^5^ cells were loaded on the silk constructs and left undisturbed in a humidified incubator (37 °C, 5% CO_2_) for 30 min for cellular adhesion. The cell laden matrices were then incubated in complete DMEM for 7 days prior to treatment with experimental drugs. The culture medium was replenished after every 2 days. 

### 2.5. Cell Proliferation Assay

The cell proliferation assay was done by seeding equal number of cells on both the 2D (1 × 10^5^) and 3D (1 × 10^5^) silk constructs. AlamarBlue, a non-toxic chemical, was used to visualize the reducing environment of the proliferating cell. This assay was executed after 1, 4, and 7 days to confirm cell viability and proliferation of the cells on 2D and 3D constructs [25].

### 2.6. Chemotherapeutic Studies

The human breast cancer cells (MDA-MB-231) were seeded on 2D (1 × 10^5^) and 3D (1 × 10^5^) culture systems separately. The cells were then incubated for 7 days in a CO_2_ (5%) chamber at 37 °C. The cell laden 2D and 3D constructs after 7 days of culture were treated with test compounds for 48 h before evaluating for cytotoxic effects by the MTT assay. The cells were serum starved for 24 h before drug treatment. The anticancer activity of cyclosaplin and doxorubicin was performed as per the National Cancer Institute (NCI) of Bethesda, MD, USA, guidelines following 10-fold dilutions of five concentrations ranging from 1–1000 µg/mL. The established chemotherapeutic agent doxorubicin served as a positive control for the study. The IC_50_ of cyclosaplin and doxorubicin were computed for the cells grown in both 2D and 3D constructs. Doxorubicin was used as a positive control as it interacts with DNA by intercalation, inhibits macromolecular biosynthesis, and is used for treating breast cancer [26,27].

### 2.7. Glucose Consumption and Lactic Acid Production Assay

The glucose present in the spent media was determined using the Glucose Assay Kit (Span Diagnostics, India). Briefly, 2µL of spent media was added to 200 µL of enzyme mixture and incubated at 37 °C for 30 min. The reaction mixture was well mixed and the absorbance was read at 505 nm using a microplate reader. All the recordings were performed in triplicates. The glucose concentration was analyzed using triplicates of a single standard (1 mg/mL) according to the manufacturer’s instructions. The amount of glucose consumption was calculated by subtraction of the total glucose present in the media with the glucose left on day 1, 4 or 7 after seeding [21]. 



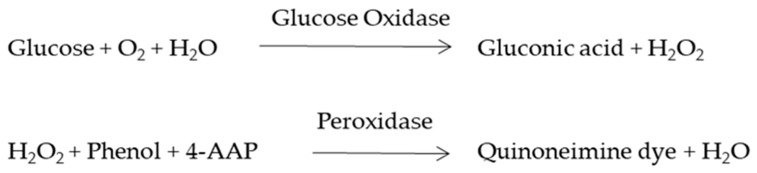



The lactate present in the spent media was determined using a lactate assay kit (Span Diagnostics, India) on grown cells for 1, 4, or 7 days. The cells were incubated at 37 °C for 3 min in the dark and then absorbance was read at 340 nm.



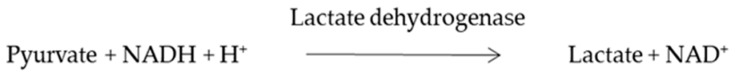



### 2.8. Matrix Metalloproteinase 9 (MMP-9) Activity of MDA-MB-231 Cells on Silk Constructs

Equal amount of cells were seeded on 2D and 3D silk constructs and were treated with or without experimental drugs at two different concentrations (1 and 100 µg/mL). MMP-9 activity in 2D and 3D silk constructs was analyzed using sodium dodecyl sulphate-polyacrylamide gel electrophoresis (SDS-PAGE) gelatin zymography. MMP-9 activity present in conditioned media was visualized by gelatin zymography as described by [28]. Gels for zymography comprised of 0.1% gelatin and 10% polyacrylamide. Samples were mixed with a Tris-HCl 0.25 M, pH 6.8, SDS 2%, sucrose 4%, bromophenol blue 0.1% buffer and electrophoresed without boiling. For qualitative analysis of gelatinase activities, 20 µg of protein from each sample was loaded onto the gel. Then gels were incubated in 2.5% Triton X-100 with gentle shaking for 30 min at 20 °C. The gels were then incubated for 18 h at 37 °C in substrate buffer (50 mM Tris–HCl, pH 7.6, 200 mM NaCl, 10 mM CaCl_2_). The gels were stained in 0.5% coomassie blue G-250 in acetic acid/methanol/water (1:4:5) for 30 min after incubation followed by destaining in acetic acid/methanol/water (1:2:7). The proteolytic activities were visualized as clear white zones, demonstrating gelatin degradation in the gels, against the stained blue background. Collagenase (Sigma, St. Louis, MO, USA) at 5 ng concentration was used as a positive control. The MMP-9 activity was analyzed according to its molecular weight (MMP-9: 92 kDa).

### 2.9. Scanning Electron Microscopy

MDA-MB-231 cells (1 × 10^5^) were seeded on 3D silk constructs. The 7th day constructs were rinsed in 500 µL of serum-free medium and fixed in 4% paraformaldehyde for 15 min. The samples were washed twice with PBS (pH 7.4), subjected to dehydration using a series of 50–100% ethanol (20 min) and vacuum-dried. For scanning electron microscopy (SEM), the images were taken after the samples were sputter coated with gold using a (JEOL JSM-5800, Tokyo, Japan) scanning electron microscope [21,23].

### 2.10. Live–Dead Assay

MDA-MB-231 cells (1 × 10^5^) were seeded on 3D silk fibroin constructs. The viability of MDA-MB-231 cells was measured using a live–dead viability kit (Invitrogen, USA) following the manufacturer’s instruction. The cultures were incubated in a humidified CO_2_ chamber for 7 days at 37 °C. The cyclosaplin and doxorubicin induced (1 µg/mL, 100 µg/mL) cell loaded silk constructs were stained with 150 μL live–dead assay reagent (2 μM Calcein AM (acetomethoxy derivate of calcein) and 4 μM ethdium homodimer-1 (EthD-1) for 30 min at 25 °C. It was then washed twice in PBS and visualized under a confocal microscope (CLSM; Olympus FV 1000 attached to an inverted microscope IX 81, Tokyo, Japan).

### 2.11. Analysis of Cytoskeletal Organization

MDA-MB-231 cells were cultured on 2D (1 × 10^3^) silk constructs as described by [29]. The cells were treated with cyclosaplin and doxorubicin. The 2% paraformaldehyde fixed cells were incubated for 15 min at room temperature, followed by permeabilization with 0.5% Triton X-100 before staining. After incubation with the tetramethylrhodamine (TRITC) conjugated phalloidin (1:200 dilution) for 1 h at room temperature, followed by rinsing with 1x PBS (pH 7.4), counter staining of the nucleus was done with Hoeschst (1:1000 dilution) for 15 min at room temperature. Imaging was done using a confocal microscope (CLSM; Olympus FV 1000 attached to an inverted microscope IX 81, Tokyo, Japan).

### 2.12. Statistical Analysis

All represented data are the mean and standard deviation of three samples. Analysis was performed using Student’s *t*-test on Graphpad Prism 7 software (San Diego, CA, USA) and the significance level of *p* < 0.05 and *p* < 0.01 was employed.

## 3. Results

### 3.1. Preparation and Fabrication of 2D/3D Silk Constructs

**Figure 1:**
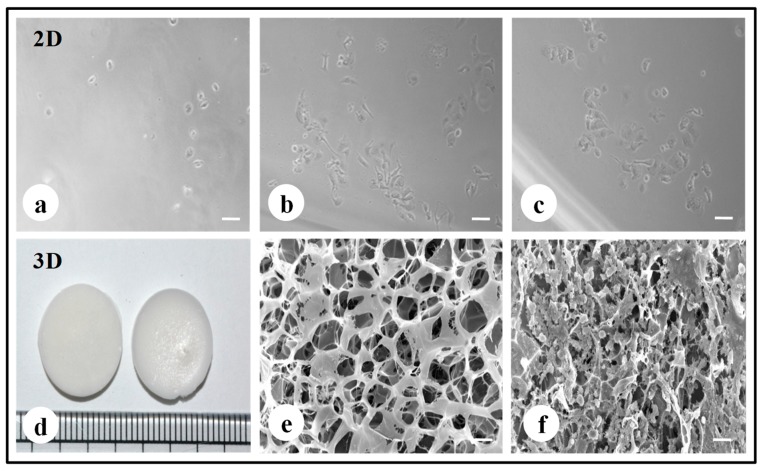
Fabrication of silk 2D and 3D silk constructs. (**a**) *A. mylitta* silk fibroin 2D construct seeded with MDA-MB-231 cells (Day 1). (**b**) *A. mylitta* silk fibroin 2D construct seeded with MDA-MB-231 cells (Day 3) (**c**) *A. mylitta* silk fibroin 2D construct seeded with MDA-MB-231 cells (Day 5). (**d**) *A.mylitta* silk fibroin constructs. (**e**) Scanning electron micrograph of *A. myliita* construct w/o cells (Scale bar = 100 µm). (**f**) *A. mylitta* fibroin construct seeded with MDA-MB-231 cells for 7 days (Scale bar = 50 µm).

The fabricated silk constructs without the cells were observed under a scanning electron microscope. The 3D silk fibroin scaffolds with interconnected pores are shown in Figure 1e. MDA-MB-231 cells propagated well on 2D fibroin coated plates within 7 days of the culture period (Figure 1a–c). MDA-MB-231 cells were grown on 3D silk constructs for 0, 1, 4, and 7 days (Figure S1). In 3D silk constructs, the MDA-MB-231 cells proliferated well, utilizing all the pores of 3D constructs forming clusters on the 7th day as shown in Figure 1f.

### 3.2. Cell Proliferation Assay

**Figure 2:**
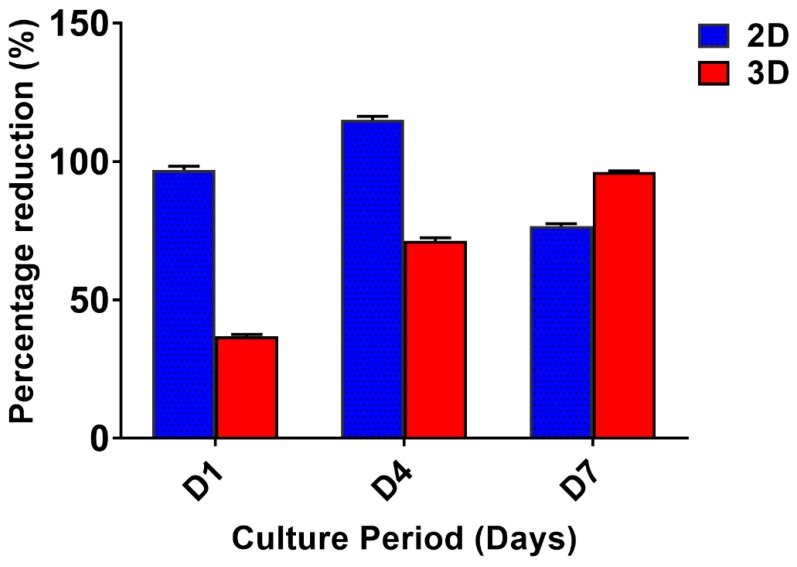
The cell proliferation of MDA-MB-231 cells grown on 2D and 3D silk fibroin constructs over a culture period of 7 days. The highest cell growth was observed on the 4th day in the 2D constructs. The proliferation of cells in 3D increased over time and was higher than 2D on the 7th day. The error bars indicate ± SD (standard deviation for *n* = 3).

The proliferation of the invasive breast cancer cells (MDA-MB-231) on 2D and 3D constructs (Figure 2) were evaluated by alamarBlue assay. The cells were proliferative over time in 3D matrices, while the peak of cell growth in 2D was revealed on the 4th day (115.2 ± 1.35%) followed by a reduction in proliferation rate. The cells in 2D have limited surface area, therefore after a certain confluence level, the viability of cells decreases due to restricted space to multiply further. The proliferation of cells in 3D increased over time and was higher than 2D on the 7th day (96.22 ± 0.40).

### 3.3. Chemotherapeutic Studies

**Figure 3:**
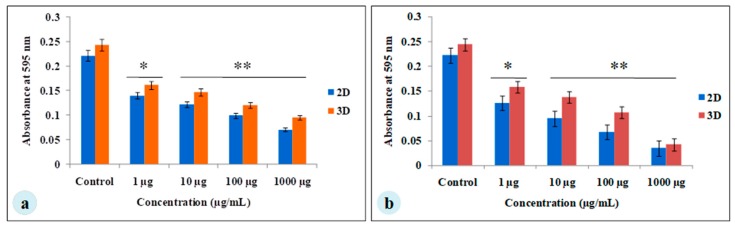
Viability of MDA-MB-231 cells treated with cyclosaplin and doxorubicin. The MDA-MB-231 cells were grown for 7 days before treatment. (**a**) Cyclosaplin (peptide) treated MDA-MB-231 cells on silk constructs. (**b**) Doxorubicin (anticancer drug) treated MDA-MB-231 cells on silk constructs. The error bars indicate ± SD (standard deviation for *n* = 3). * *P* < 0.05; ** *P* < 0.01.

The MDA-MB-231 cells were cultured for 7 days on *A. myliita* silk constructs in vitro, and subjected to cyclosaplin and doxorubicin treatment for 48 h. The cell viability was studied for various drug concentrations (1–1000 µg/mL) of the peptide (Figure 3a) and doxorubicin (Figure 3b). In 2D culture, the IC_50_ concentration for doxorubicin was 2.8 ± 0.06 μg/mL, whereas the IC_50_ of cyclosaplin was 16.86 ± 0.09 μg/mL. In 3D culture, the IC_50_ concentration for doxorubicin was 16.44 ± 0.1 μg/mL, while the IC_50_ of cyclosaplin was 89.27 ± 0.2 μg/mL. The higher concentrations of cyclosaplin and doxorubicin were needed to achieve the IC_50_ value in human breast cancer cells grown in 3D (Table 1).

### 3.4. Glucose Consumption and Lactate Dehydrogenase Assay

**Figure 4:**
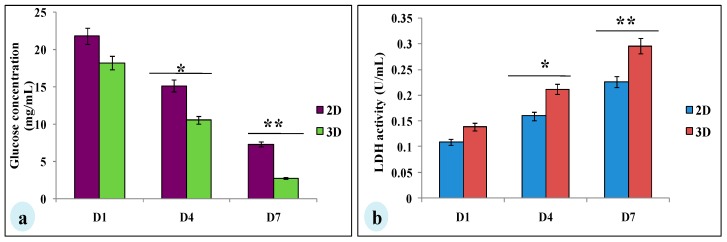
Glucose metabolic studies of MDA-MB-231 cells cultured on 2D and 3D silk constructs; (**a**) glucose concentration (mg/mL) and (**b**) LDH activity (U/mL) in the spent media of cells grown on 2D and 3D constructs. The data is denoted as mean ± SD (*n* = 3, *P* < 0.01, *P* < 0.05). * *P* < 0.05; ** *P* < 0.01.

To evaluate whether glucose metabolism of MDA-MB-231 cells is altered in silk constructs, the collected spent media on 1, 4, or 7 days after seeding and glucose concentration was measured. In 3D silk constructs, the glucose concentration on the 7th day was 2.76 ± 0.03 mg/mL, compared to 2D (7.3 ± 0.08 mg/mL). In 3D silk constructs, a 1.3-fold increase in LDH activity was observed on the 7th day in comparison to 2D. Thus, the glucose consumed and LDH activity was higher in cells grown in 3D constructs than those in 2D (Figure 4).

### 3.5. MMP-9 Activity of MDA-MB-231 Cells on Silk Constructs

**Figure 5:**
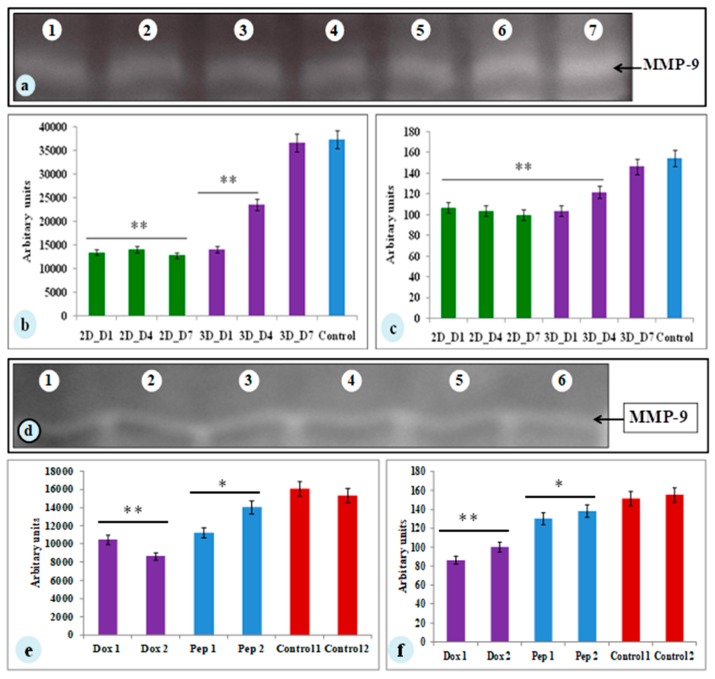
The zymograph of spent media from MDA-MB-231 cells cultured on constructs. (**a**) Lane 1– Lane 3: 2D *A. mylitta* coated silk constructs (Day 1, Day 4, Day 7), Lane 4–Lane 6: 3D *A. mylitta* silk fibroin scaffolds, Lane 7: collagenase (positive control). Bands were analyzed using Image J –NIH software and represented as (**b**) mean area and (**c**) mean intensity (arbitrary units). Zymograph of drug treated spent media from MDA-MB-231 cells grown on 3D constructs. (**d**) Lane 1–Lane 2: doxorubicin treated (1 and 100 µg/mL) *A. mylitta* silk fibroin constructs, Lane 3–Lane 4: cyclosaplin treated (1 and 100 µg/mL) *A. mylitta* silk fibroin constructs, Lane 5: control, and Lane 6: collagenase (positive control). Bands were analyzed using Image J software and represented as (**e**) mean area and (**f**) mean intensity (arbitrary units). * *P* < 0.05; ** *P* < 0.01.; Dox—Doxorubicin; Pep—Peptide, Cyclosaplin.

MMP-9 plays a vital part in the progression of tumor and angiogenesis. To determine the MMP-9 activity, SDS-PAGE gelatin zymography was performed. The spent media of day 1, 4, and 7 of both the constructs (2D and 3D) were assessed for MMP-9 activity (Figure 5). MMP-9 activity was observed in both the 2D and 3D cell cultures. The cells cultured on 3D silk constructs showed higher activity (1.4-fold increase) on the 7th day compared to 2D cultures (Figure 5a–c). The 3D silk constructs were therefore, used for the experimental drugs studies. The spent media of the cyclosaplin and doxorubicin (1, 100 µg/mL) treated 3D constructs were evaluated for MMP-9 activity. The cyclosaplin and doxorubicin treated MDA-MB-231 cells showed a 1.2- and 1.5-fold decrease in MMP-9 activity in 3D constructs, respectively (Figure 5d–f).

### 3.6. Scanning Electron Microscopy

**Figure 6:**
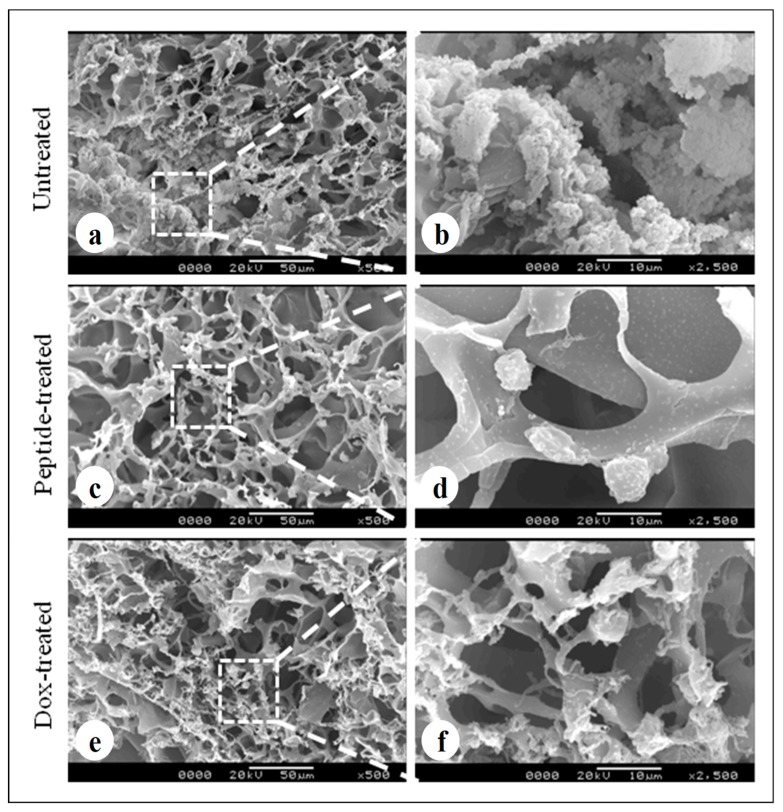
Cell loaded 3D silk constructs displaying treated and untreated conditions: (**a**,**b**) MDA-MB-231 cells loaded silk fibroin construct without treatment, (**c**,**d**) peptide treated (cyclosaplin, 100 µg/mL) MDA-MB-231 cells on *A. mylitta* silk fibroin, (**e**,**f**) doxorubicin (100 µg/mL) treated MDA-MB-231 cells on *A. mylitta* silk fibroin. Scale bar = 50 and 10 µm.

The scanning electron micrographs revealed formation of cellular aggregates, distributed homogenously within the 3D silk constructs after a culture period of 7 days in untreated constructs (Figure 6a). Both cyclosaplin (peptide) (Figure 6c,d) and doxorubicin (Figure 6e,f) treated (100 µg/mL) constructs exhibited few individual cells that were less in number. Cell shrinkage was also observed in both the treated conditions.

### 3.7. Live–Dead Assay

**Figure 7:**
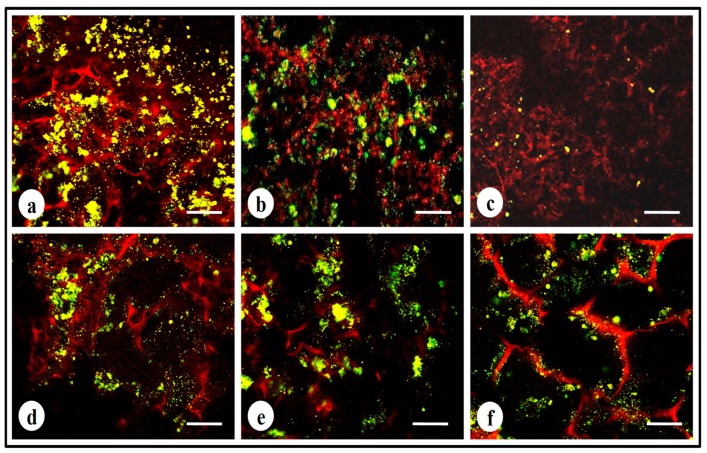
Live–dead stained confocal micrographs showing morphology of MDA-MB-231 cells: (**a**,**d**) untreated MDA-MB-231 cells, (**b**) doxorubicin treated cells (1 µg/mL), (**c**) doxorubicin treated cells (100 µg/mL), (**e**,**f**) cyclosaplin treated cells (1 and 100 µg/mL). Scale bar = 200 µm.

To study the cell viability of MDA-MB-231 cells after treatment with cyclosaplin (1, 100 µg/mL) and doxorubicin (1, 100 µg/mL), a live–dead assay was performed. The number of viable cells decreased in the cyclosaplin and doxorubicin treated cell cultures. The number of live cells was higher in the control silk constructs (Figure 7a). Some dead cells were also observed in the doxorubicin treated culture (Figure 7b,c). The drug induced MDA-MB-231 cells reduced in size displaying unhealthy cell morphometric features (Figure 7).

### 3.8. Analysis of Cytoskeletal Organization

**Figure 8:**
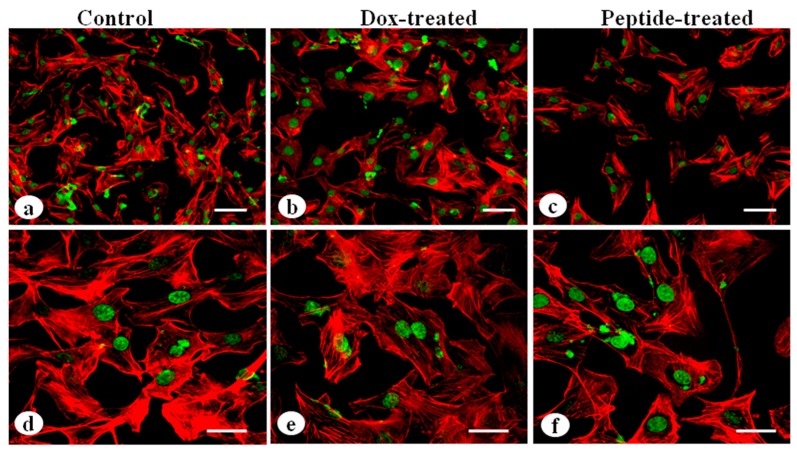
Cytoskeletal organization of MDA-MB-231 cells by actin immunostaining: (**a**,**d**) MDA-MB-231 cells without treatment, (**b**,**e**) MDA-MB-231 cells treated with doxorubicin, (**c**,**f**) cyclosaplin treated MDA-MB-231 cells (Scale bar = 50 µm and 20 µm).

The MDA-MB-231 cells grown on 2D silk constructs were immunostained with actin antibody to visualize the effects of cyclosaplin and doxorubicin on cytoskeletal arrangements. The cyclosaplin and doxorubicin treated cells showed some membrane ruffles and a decrease of cytoskeletal stress fibers (Figure 8). No such significant changes in cytoskeletal organization were observed in both the cases.

## 4. Discussion

In this study, a 3D breast tumor model was used for culturing MDA-MB-231 cells on *A. mylitta* silk fibroin and screened for efficacy of the experimental anticancer drugs. The objective of this study was to investigate whether efficacy of cyclosaplin and doxorubicin are affected when screened in cells cultured on 3D silk constructs compared to conventional 2D systems prior to future preclinical trials. 

For the experimental studies, the *A. mylitta* silk constructs were fabricated [21] to facilitate the attachment and proliferation of human breast cancer cells (MDA-MB-231). The RGD peptide serves as a recognition site for integrin-mediated cell adhesion present in *A. myllita* fibroin and plays a vital role in cell attachment and proliferation [29,30]. Scanning electron micrographs revealed the well-interconnected pores ranging from 100–300 µm, as well as proliferating MDA-MB-231 cells growing in clusters (Figure 1). Previous research has indicated similar cancer cell phenotypes in 3D models, which is indicative of tumors in vivo [10,31]. AlamarBlue, a redox indicator was used to assess the metabolic function and cellular health of the proliferating cells. The cellular growth of cells was higher in the 3D constructs compared to 2D constructs. MDA-MB-231 cells grown in 2D constructs achieve 80% confluency on the 4th day, in contrast to 3D silk constructs. The initial growth of cells was slow in 3D silk constructs but increased on the 7th day (Figure 2). The slow initial growth of cells in 3D culture compared to corresponding 2D culture was in support of earlier reports that describe the difference in cell signaling and cellular functions between 3D and 2D responsible for such phenomena [22]. After initial cell seeding in 3D, cell adhesion organization develops in vitro towards in-vivo-like adhesions [5] that impart a reduced cellular proliferation rate during the initial culture period. Also, the cellular growth in 3D constructs recapitulates the mathematical model of tumors in vivo better than 2D constructs. The cell viability of MDA-MB-231 cells after treatment with cyclosaplin and doxorubicin were investigated using the MTT assay (Figure 3a,b). The 3D silk fibroin constructs showed a higher density of cells than that of the 2D constructs. For the two experimental drugs, cyclosaplin and doxorubicin, the IC_50_ values were 5–6 fold higher than in the 2D monolayer (Table 1). This suggests that MDA-MB-231 cells in 3D architecture have a higher perquisite of concentration of experimental drugs to achieve 50% cell death. Earlier, anticancer drugs such as doxorubicin, paclitaxel, and tamoxifen demonstrated 12–23-fold higher IC_50_ values in a 3D breast (MCF-7) cancer model compared to 2D systems [32]. Similarly, celexocib, ZD6474, and paclitaxel showed 2–6 fold higher IC_50_ values in a 3D silk fibroin tumor model [22]. The higher number of cells could be responsible for increased resistance towards anticancer drugs. The tumor matrix and cells in the tumor might be altering the response of the tumor cells towards the drugs. The complex biological processes involved in the tumor microenvironment partly define its phenotype, including resistance to drugs [33]. Several researchers have spotlighted the role of 3D cell culture in high resistance to drugs [34,35,36,37,38]. Recently, high-throughput 3D evaluation revealed variation in drug sensitivities between culture models of JIMT1 breast cancer cells [39]. It has long been postulated however that cells grown in 3D matrices can show relative resistance to chemotherapeutic drugs and mimic in vivo like conditions, absent in 2D culture systems [40]. Further, glucose metabolism of MDA-MB-231 cells was investigated on 2D and 3D silk constructs. The glucose consumption and LDH activity was significantly higher in 3D constructs compared to 2D (Figure 4a,b). This phenomenon was comparable to earlier reports that describe high glucose consumption and lactic acid production in 3D constructs [21]. Cancer cells ardently use glucose as an energy source and for survival in comparison to normal cells [41]. It is observed that cancer cells metabolize glucose into pyruvate, producing excess lactate even though there is an adequate supply of oxygen to support mitochondrial respiration [42,43]. An elevated level of LDH is a hallmark of several highly glycolytic tumors and leads to a poor prognosis in several cancers [44,45]. Researchers have shown a positive correlation between lactate levels and tumor burden in cancer patients [44,45]. Thus, it was postulated that the enhanced levels of LDH and the production of lactic acid by tumor cells might facilitate escape from immune surveillance [45,46,47]. Several researchers have comparatively evaluated the glucose metabolism in both 2D and 3D tumor models in vitro [21,47]. The MMP-9 activity was higher in 3D cell cultures than 2D, suggesting a high proliferative rate, survival, and invasive nature of the breast cancer cells (Figure 5a–c). Matrix metalloproteinases have long been connected with cancer-cell invasion and metastasis [14]. MMPs regulate signaling pathways that control cell growth, survival, invasion, inflammation and angiogenesis [48]. The cyclosaplin and doxorubicin treated MDA-MB-231 cells however, showed a 1.2–1.5-fold decrease in MMP-9 activity (Figure 5d–f). Thus, the decrease in MMP-9 activity suggests the usefulness of cyclosaplin as a MMP-9 inhibitor. MMP-9 has been found in large quantities in the cancer tissues and corroborates with the process of tumor cell invasion and metastasis [49,50]. Recent clinical studies in cancer patients, have implicated that MMP-9 could be a strong and independent marker for aggressive breast cancer [51]. Apart from MMP-9, evidence indicates higher expression of VEGF, interleukin 8, and protein kinase C in 3D constructs compared to 2D constructs [1]. Recently, gene expression studies of selected genes involved in breast cancer progression was assessed in heterogenic 3D tumor models [2]. Scanning electron micrographs displayed the cytotoxic effect of cyclosaplin and doxorubicin on MDA-MB-231 cells. The number of cells reduced after treatment with the experimental drugs in 3D cell culture and showed unusual morphology (Figure 6). The shrinkage of cells post-treatment with experimental drugs may be due to apoptosis. In our previous studies, we have shown the anti-proliferative and apoptotic role of cyclosaplin on MDA-MB-231 cells (2D cultures) by activating caspase-3 [21]. Similarly, the live–dead assay showed the detrimental effects of doxorubicin (Figure 7b,c) and cyclosaplin (Figure 7d–f) on MDA-MB-231 cells, although a higher concentration was a prerequisite to achieve 50% cell death in 3D silk constructs (Figure 7). The MDA-MB-231 cells (2D culture) treated for 24 h with cyclosaplin and doxorubicin apparently did not modify the MDA-MB-231 actin cytoskeleton but a few membrane ruffles were observed in the cyclosaplin treated cells (Figure 8). The presence of a few membrane ruffles indicates a connection between actin re-arrangement and deregulation of adhesion. However, further studies are essential to explore the role of cyclosaplin as an actin targeting drug. The targeting of the actin cytoskeleton is quite old, given its involvement in cell-related processes such as cell division and cell migration [52,53]. The actin cytoskeleton, therefore, is one of the target points for cancer therapeutics. TR100, a novel class of anti-tropomyosin compounds, is active against a panel of neural crest-derived tumor cell lines in both 2D and 3D cultures with minimum effect on the contractile features of isolated rat adult cardiomyocytes [54]. Previously, the other actin targeting drugs, such as cytochalasin D and jasplakinolide, have shown huge potential as antiproliferative agents in vitro [55,56]. Several researchers have reported the different possibilities as to how the 3D microenvironment may influence proliferation, presence or absence of growth factors, cytokines, proteases, or alterations in the composition or structure of ECM proteins [5,8,56]. 

Our study indicates the potential of cyclosaplin, a cyclic octapeptide as an anti-cancer agent against breast cancer, however further experimental studies demonstrating an alteration in proliferation (cyclin B1, E2F1, Ki-67, and proliferating cell nuclear antigen), angiogenesis (VEGFA), and apoptosis markers (Caspase 3, Bax) is required in the 3D silk tumor model. Apart from breast cancer, the effect of cyclosaplin on other cancer cell types, such as colon, cervical, and melanoma, can be studied in 3D silk tumor models.

## 5. Conclusions

We assessed the efficacy of cyclosaplin, a cyclic octapeptide on a 3D silk fibroin in vitro tumor model. Cyclosaplin altered the proliferative, survival, invasive, and metabolic capacity of MDA-MB-231 cells in 3D cell cultures compared to a 2D cell monolayer. The heterogeneity of tumor cells in vivo can be recapitulated better by culturing breast cancer cells with other cell types in 3D models for high-throughput screening of peptide-based drugs. Overall, the results throw a spotlight on the usefulness of cyclosaplin in cancer therapeutics.

## Figures and Tables

**Table 1:** Chemotherapeutic studies of cyclosaplin and doxorubicin.

Drug	IC _50_ (μg/mL)		Fold Change in IC_50_
	2D Monolayer	3D Model	
Cyclosaplin	16.8 6 ± 0.09	89.27 ± 0.2	5.3
Doxorubicin	2.8 ± 0.06	16.44 ± 0.1	5.9

## Supplementary Materials

The following are available online at https://www.mdpi.com/2218-273X/9/4/123/s1. Figure S1: Fabrication of silk 3D constructs.
