# Supplementary material for: Evaluation of Cyclosaplin Efficacy Using a Silk Based 3D Tumor Model
Source: Biomolecules. 2019 Mar 28;9(4):123. doi: 10.3390/biom9040123 (PMC6523308; doi:10.3390/biom9040123)
Supplement: Supplementary file 1 [file biomolecules-09-00123-s001.pdf]

Figure S1

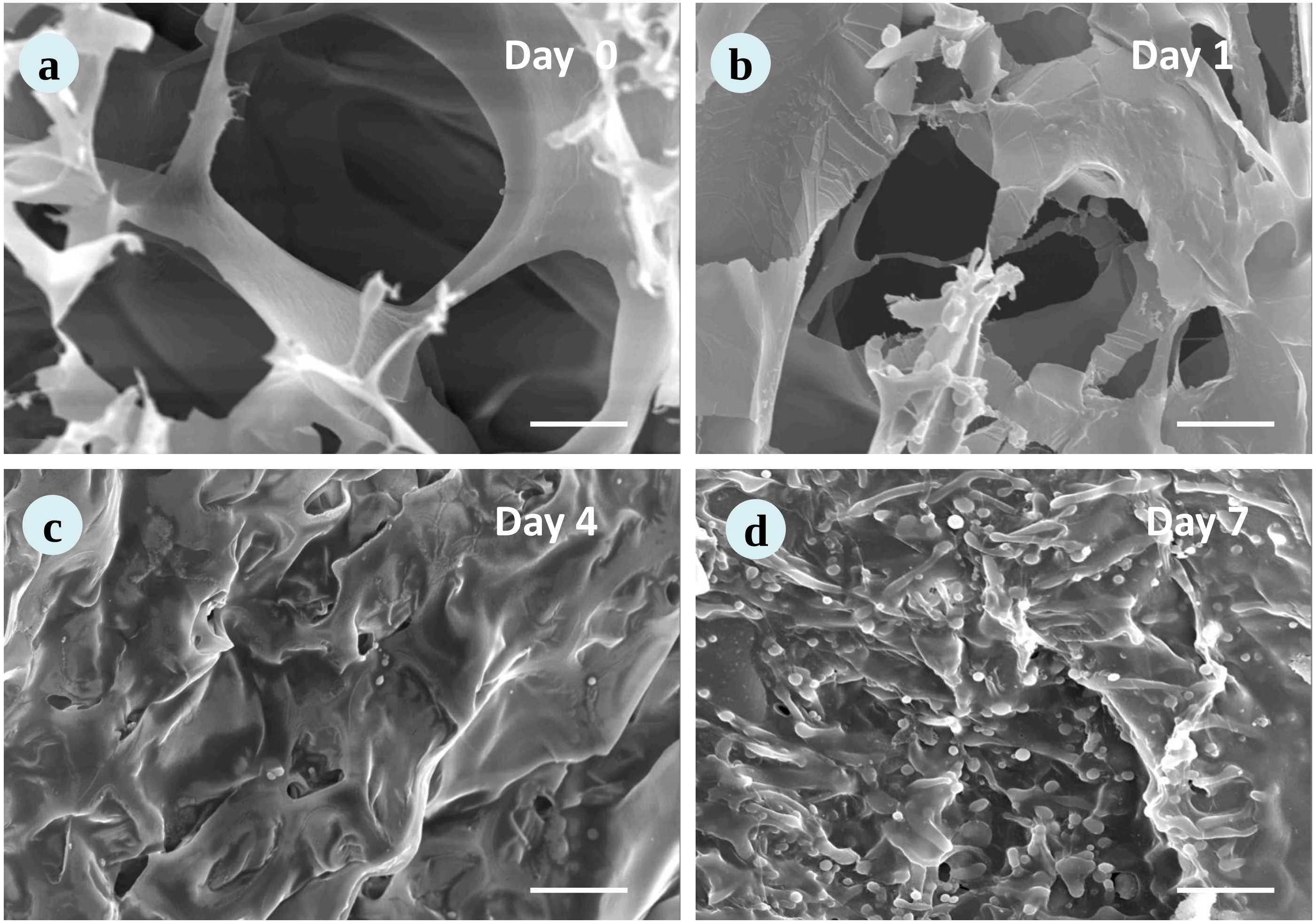

Fabrication of silk 3D Constructs. a) – d) MDA-MB-231 cells were grown on 3D silk constructs for 0, 1, 4, and 7 days
